# *Crotalus durissus terrificus* crotapotin naturally displays preferred positions for amino acid substitutions

**DOI:** 10.1186/s40409-017-0136-5

**Published:** 2017-11-28

**Authors:** Laudicéia Alves de Oliveira, Rui Seabra Ferreira, Benedito Barraviera, Francilene Capel Tavares de Carvalho, Luciana Curtolo de Barros, Lucilene Delazari dos Santos, Daniel Carvalho Pimenta

**Affiliations:** 1 Postgraduate Program in Tropical Diseases, Botucatu Medical School, Sao Paulo State University (UNESP), Botucatu, SP Brazil; 2 Center for the Studies of Venoms and Venomous Animals (CEVAP), São Paulo State University (UNESP), Botucatu, SP Brazil; 3 Laboratory of Biochemistry and Biophysics, Butantan Institute, Av. Vital Brazil, 1500, São Paulo, SP CEP 05503-900 Brazil

**Keywords:** *Crotalus durissus terrificus*, Venom, Crotoxin, Crotapotin, Isoforms

## Abstract

**Background:**

Classically, *Crotalus durissus terrificus* (Cdt) venom can be described, according to chromatographic criteria, as a simple venom, composed of four major toxins, namely: gyroxin, crotamine, crotoxin and convulxin. Crotoxin is a non-covalent heterodimeric neurotoxin constituted of two subunits: an active phospholipase A_2_ and a chaperone protein, termed crotapotin. This molecule is composed of three peptide chains connected by seven disulfide bridges. Naturally occurring variants/isoforms of either crotoxin or crotapotin itself have already been reported.

**Methods:**

The crude Cdt venom was separated by using RP-HPLC and the toxins were identified by mass spectrometry (MS). Crotapotin was purified, reduced and alkylated in order to separate the peptide chains that were further analyzed by mass spectrometry and de novo peptide sequencing.

**Results:**

The RP-HPLC profile of the isolated crotapotin chains already indicated that the α chain would present isoforms, which was corroborated by the MS and tandem mass spectrometry analyses.

**Conclusion:**

It was possible to observe that the Cdt crotapotin displays a preferred amino acid substitution pattern present in the α chain, at positions 31 and 40. Moreover, substitutions could also be observed in β and γ chains (one for each). The combinations of these four different peptides, with the already described chains, would produce ten different crotapotins, which is compatible to our previous observations for the Cdt venom.

## Background

Snake venoms are complex mixtures rich in proteins and peptides, in which such molecules can comprise up to 95% of the venom dry weight [1, 2]. Such molecules do aid the animal survival, once they may be used either as a hunting tool or as a defense mechanism [3]. Moreover, these toxins are also involved in ophidian accidents [4]. Crotoxin, the major *Crotalus durissus terrificus* (Cdt) venom toxin, is also the most toxic [5, 6]. It is a heterodimeric neurotoxin comprised of a basic phospholipase A_2_ (PLA_2_) and an acidic protein, also known as crotapotin [7, 8].

Crotapotin, a 9.6-kDa peptide displaying a pI of 3.4, was initially characterized as a chaperone since the PLA_2_ would increase its toxicity and inhibit the PLA_2_ activity [9–12]. However, this peptide has also been described as presenting anti-inflammatory activity and being able to modulate the humoral immunity, including in some neurodegenerative autoimmune disorders [13–17].

Structurally, crotapotin is composed of three peptide chains, connected by seven disulfide bonds [16, 18]. These chains, called α, β and γ, were first sequenced in 1985 and determined to be composed of 40 (α-chain), 35 (β-chain) and 14 (γ-chain) amino acids [19, 20]. However, some authors have observed the occurrence of natural variations of crotapotin [17, 21].

In the present study, we have developed a method for the isolation and biochemical characterization of crotapotin from crude Cdt venom, including the chromatographic separation of the peptide chains after reduction and alkylation, and de novo mass spectrometry peptide sequencing.

## Methods

### Venoms and animals

Pooled Cdt venom was obtained from snakes kept in the Center for the Studies of Venoms and Venomous Animals (CEVAP) of UNESP, in Botucatu (Brazil). All procedures involving snake specimens were in accordance with the ethical standards of the institutional and/or national research committee. The study was approved by the responsible Ethics Committee on Animal Use of Botucatu Medical School (protocol n^o^ 1145/2015 – CEUA).

### RP-HPCL

A 10 mg.mL^−1^ crude Cdt venom solution (0.1% trifluoroacetic acid – TFA) was centrifuged (3800 x g) and separated by RP-HPLC using a Luna C8 column (100 A, 250 × 10 mm, Phenomenex) coupled to a Shimadzu Proeminence binary HPLC system. A 20–40% linear gradient of B (90% acetonitrile – ACN, containing 0.1% TFA) over A (0.1% TFA) was used for 40 min after initial isocratic elution for 5 min, under a constant flow of 5 mL.min^−1^. UV monitoring was performed at 214 nm and fractions were manually collected. The reduced and alkylated crotapotin chains were separated by a Shimpack C18 column (100 A, 10 × 4.6 mm, Shimadzu), using a 0–50% linear gradient of B, for 20 min, under constant flow of 1 mL.min^−1^. UV monitoring was performed at 225 nm.

### Chemical processing

Isolated crotapotin was reduced with 500 mM DTT in 50 mM NH_4_HCOOH for 25 min at 56 °C and alkylated with 500 mM IAA for 30 min, at 25 °C, protected from light. The isolated α and β chains were chemically hydrolyzed with 70% formic acid for 48 h, at 37 °C. Reaction was stopped by water addition followed by lyophilization.

### Mass spectrometry and de novo peptide sequencing

A Bruker ESI-Q-TOF instrument, coupled to a Prominence Shimadzu binary HPLC, was employed for MS and MS/MS experiments. Samples were placed in the autosampler holder and submitted to a 10–80% linear gradient of B for 15 min, under constant flow of 0.2 mL.min^−1^, using a Shimpack C18 column (100 A, 10 × 2 mm). CID fragmentation for MS/MS experiments was performed with N_2_. Data were acquired under a 50–2000 m/z window and processed by Peaks Studio Suite.

## Results

### Crotapotin isolation

**Fig. 1:**
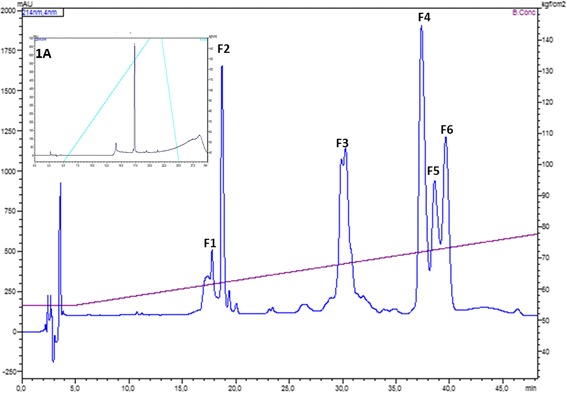
RP-HPLC profile of the crude Cdt venom. F1 to F6 correspond to the manually collected fractions. F1 and F2: crotamin; F3: crotapotin; F4, F5 and F6: PLA_2_. UV monitoring 214 nm. Inset: F3 analytical RP-HPLC demonstrating the proper molecule isolation. Chromatographic conditions are described in Methods section

**Fig. 2:**
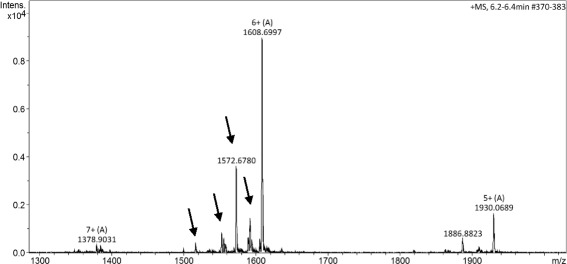
F3 ESI^+^ MS spectrum. The charge states of the major ions are presented above the m/z value. The presence of isoforms is indicated by the arrows for the [M + 6H]^6+^ ion

Figure 1 presents the C8-RP-HPLC profile of the crude Cdt venom separated according to the Methods section. Six fractions (F1-F6) were manually collected and submitted to MS analyses in order to identify the known toxins. F1 and F2 are crotamins, F3 corresponds to crotapotin and F4, F5 and F6 are PLA_2_s. The minor peaks were not collected or analyzed by MS. F3 was then submitted to another chromatographic step (Fig. 1, inset) in order to assess its homogeneity and the molecular mass was determined (Fig. 2). Figure 2 already points out to the presence of more than one molecule; however, the charge states are only indicated for the major ions.

### Crotapotin chains isolation

**Fig. 3:**
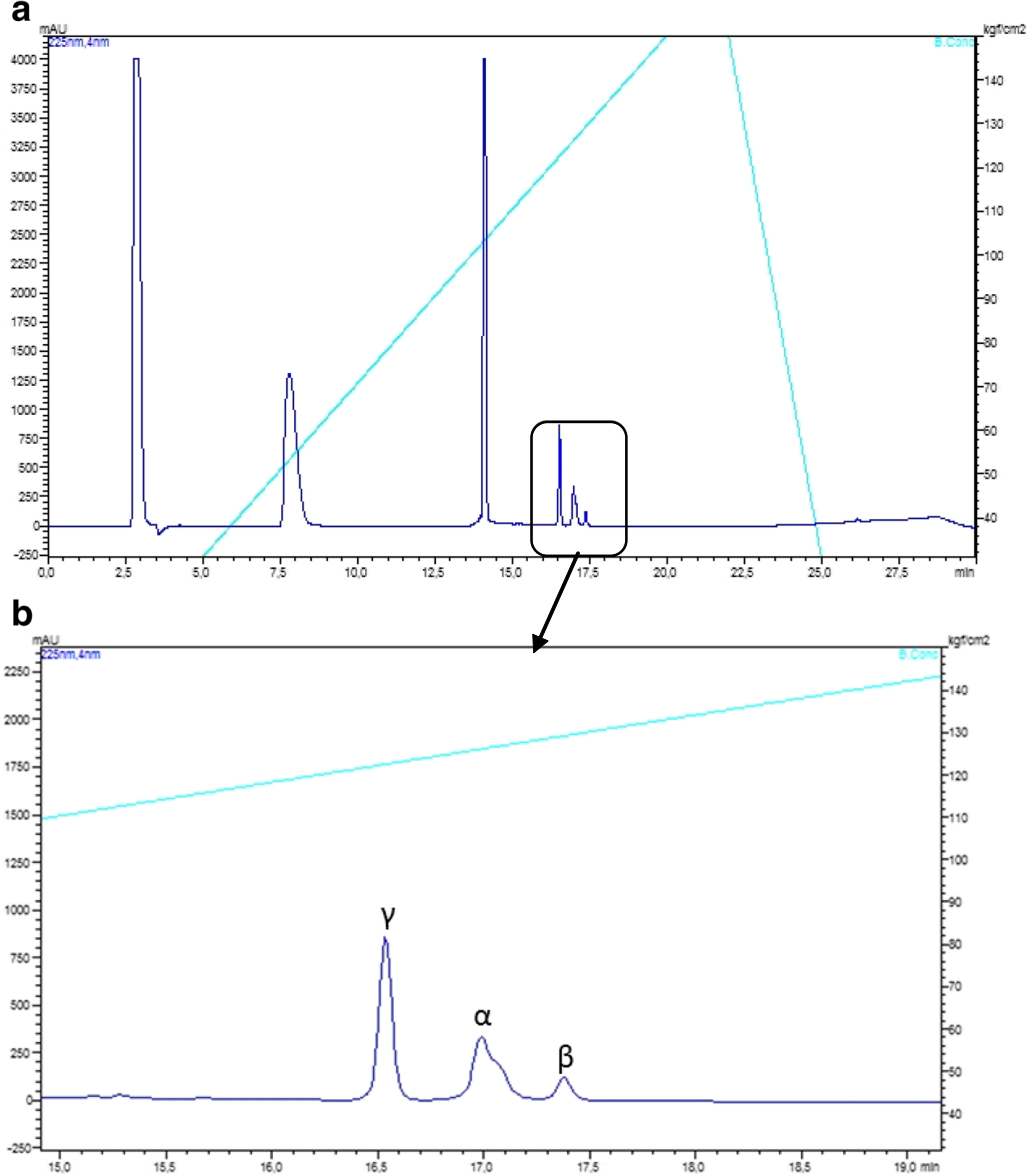
**a** Reduced and alkylated crotapotin (F3) RP-HPLC separation chromatographic profile. **b** Zoomed region with the identification of the individual chains. UV monitoring 225 nm. The major peaks in A correspond to the alkylation reagents

**Fig. 4:**
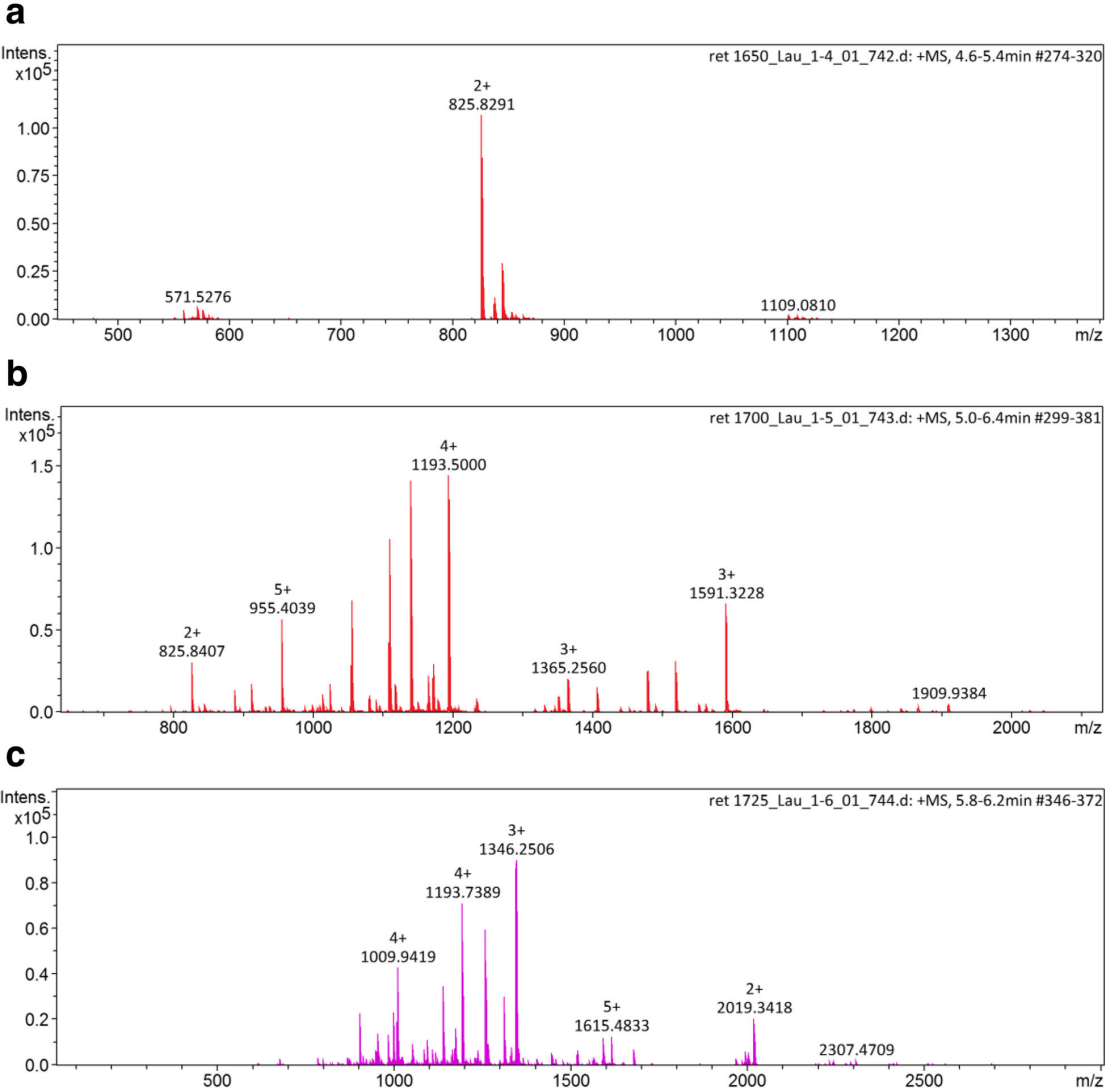
MS spectrum of the (**a**) γ, (**b**) β and (**c**) α chains. The charge states of the major ions are presented above the m/z value. The lack of homogeneity indicates the presence of isoforms

Following reduction and alkylation, as described in Methods, the processed crotapotin was subjected to RP-HPLC chromatographic separation in order to obtain the isolated α, β and γ chains. Figure 3a and b (zoomed chromatogram) present the chain separation. Major peaks in the chromatogram correspond to the reagents (data not shown). The chains were identified based on their molecular masses, as presented in Fig. 4a–c.

### De novo peptide sequencing

**Fig. 5:**
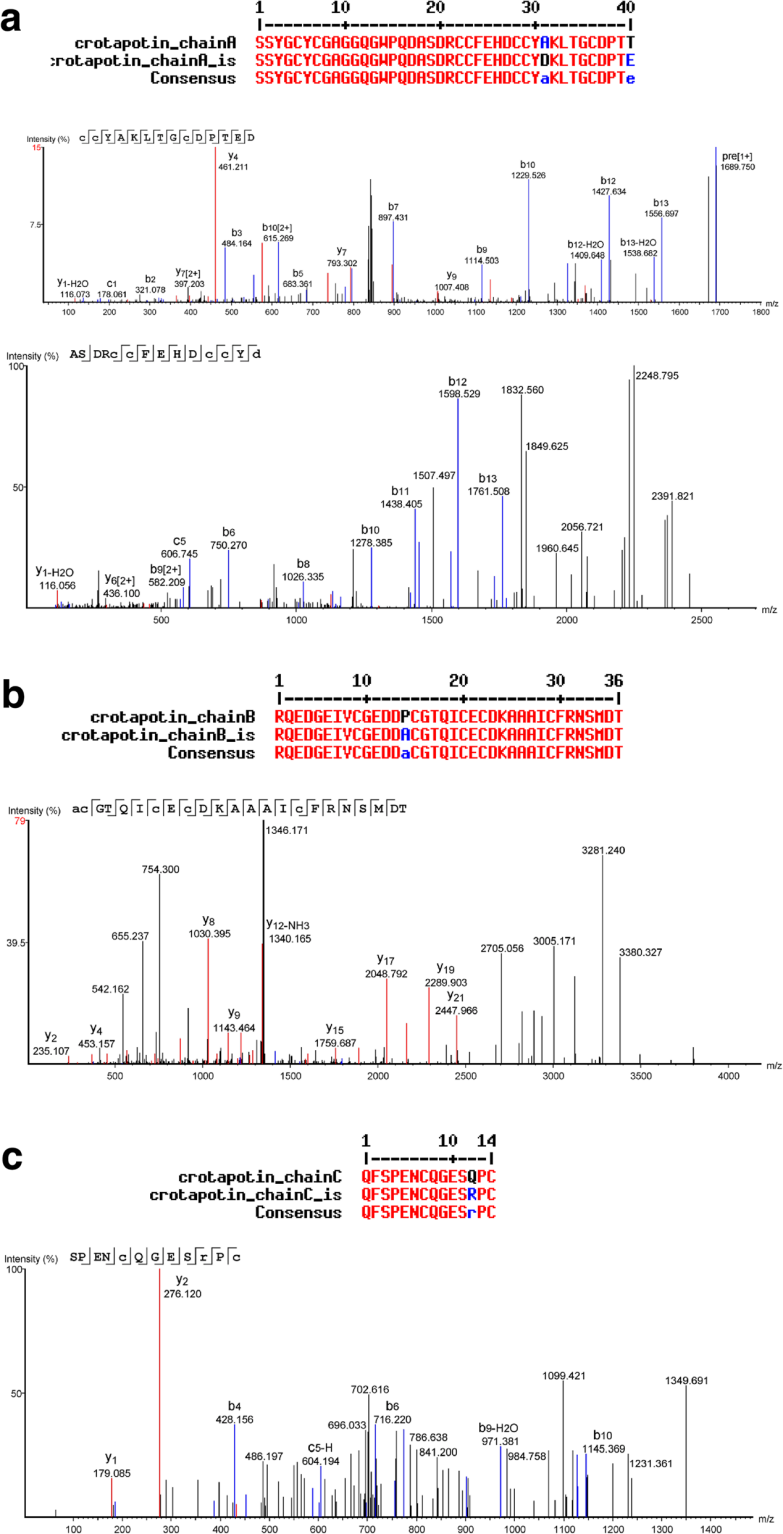
Representative annotated interpreted CID fragmentation spectra of the de novo sequenced isoforms of crotapotin (**a**) α, (**b**) β and (**c**) γ chains. Above each chain, the aligned sequences presenting the amino acid substitution are shown

The isolated formic acid hydrolyzed crotapotin chains were analyzed by LC-MS/MS (ESI-Q-TOF) and the fragmentation spectra are presented in Fig. 5a, b and c. Only the spectra of the isoforms are presented. Several spectra, corresponding to the known/deposited sequences, were obtained, but are not presented.

## Discussion

Crotoxin is a β neurotoxin, composed of two subunits: an active PLA_2_ and the catalytic inactive crotapotin [22, 23]. Since most venoms and toxins present isoforms, a consequence of an evolutionary strategy, we have chosen to evaluate whether there would be a preferred substitution site in a given crotapotin subunit that would give rise to the previously observed isoforms [21].

In order to achieve this goal, we developed a chromatographic method for the separation of crotapotin from the crude Cdt venom, and another method for the obtainment of the reduced and alkylated subunits. Moreover, due to the particular amino acid composition of the subunits, classical proteomics approaches (based on trypsin hydrolysis) could not be performed. Conversely, we have opted for the formic acid hydrolysis to adjust the peptide size to the CID fragmentation requirements. Interestingly, such strategy helped in the identification of a particular α-chain isoform, in which the deposited Ala^31^ residue was replaced by an Asp, yielding a new formic acid cleavage site that was successfully used in the de novo sequencing process (Fig. 5a).

According to our analyses, what happens in the venom gland is merely amino acid substitutions and not alternate processing, i.e., we were not able to detecte longer or shorter chains, only isoforms regarding residue changes [9]. Most of the isoforms did present molecular masses close to the already known molecule [16].

During the course of the work, we successfully covered 97% of crotapotin using the proteomics/de novo sequencing (data not shown). However, few spectra did not match the deposited sequence and, by using a combination of the Spider algorithm of Peaks Studio and manually checking the spectra for correction, we were able to identify four amino acid substitutions. Interestingly, the α chain bears more amino acid substitutions, as the asymmetrical HPLC peak already indicated (Fig. 3b). Since the α and β chains are homologous to the PLA_2_, such preference for mutation in these chains may have a counter part in the isoforms also observed for the Cdt PLA_2_ itself [21, 24].

Our de novo data specifically indicates the Thr → Glu^77^, Ala → Asp^68^ substitutions in the α chain. Although the Thr → Glu^77^ substitution would retain the hydrogen bond capabilities (but with the addition of a true charge), the Ala → Asp^68^ substitution seems more disturbing. Such mutation is located in an α helix and the introduction of a charged residue may alter such structure. Moreover, this particular region is mostly involved in the PLA_2_ interaction [23].

In the β chain, the Pro → Ala^98^ substitution would evoke the same type of alteration. Proline is a rigid, structurally relevant amino acid, typically present in protein ‘turn’ regions. On the other hand, alanine is a much more flexible amino acid. Such substitution would relax this molecule region, allowing for more flexibility and, therefore, different types of intermolecular interactions.

The γ chain (also known as crotalphine [25]) presents a Gln → Arg^136^ substitution. This modification has already been reported by Konno et al. [25] and is described as not capable of altering the analgesic properties of this peptide. This is in agreement with our proposal based on the conservation of the electrostatic characteristics of the residue that would retain the hydrogen bond-forming capabilities, in spite of the addition of a charge.

Unfortunately, the absence of genomic (or transcriptomic) data makes it more difficult to characterize other amino acid substitutions that are certainly occurring but, due to the lower relative concentration levels, have not yielded high quality spectra, suitable for the de novo sequencing (data not shown). Few studies report other amino acid substitutions, such as residue 84 of the β chain [26].

Evolution has long been ‘experimenting’ with amino acids substitutions in proteins and peptides in order to increase venom efficiency and efficacy as well as avoid prey evasive strategies [27, 28]. Not only that, but also synergism aroused by the presence of several isoforms of a given molecule also increases toxicity [29]. If one takes into account the biotechnological appeal of the clinical use of toxins, such isoforms may hint to specific targets when given residues in the toxins are substituted; the *Conus* toxins are a good example of this concept [30].

## Conclusion

The mere existence of venoms and toxic animal secretions is itself a demonstration of an unbalanced (or biased) evolutionary strategy happening in a given organism. Toxins are molecules that must act in another organism, and not against the producing animal. Therefore, regulation mechanisms must exist to prevent local damage. They may include, but are not limited to, the presence of concentrated low affinity inhibitors; the absence of ionic co-factors; the lack of catalytic activity due to conformation restrains; the pH of the media; the timing of the activation of the precursor, among others. Certainly, one of those strategies targeted to increase the efficiency and efficacy of the venom is the presence of isoforms of a given toxin, as herein reported.

## Abbreviations

ACN: Acetonitrile
Cdt: *Crotalus durissus terrificus*
MS: Mass spectrometry
MS/MS: Tandem mass spectrometry
PLA_2_: Phospholipase A_2_
TFA: Trifluoroacetic acid
